# Diversity and adaptive evolution of *Saccharomyces* wine yeast: a review

**DOI:** 10.1093/femsyr/fov067

**Published:** 2015-07-23

**Authors:** Souhir Marsit, Sylvie Dequin

**Affiliations:** 1 INRA, UMR1083, SPO, F-34060 Montpellier, France; 2 Montpellier SupAgro, UMR1083, SPO, F-34060 Montpellier, France; 3 Montpellier University, UMR1083, SPO, F-34060 Montpellier, France

**Keywords:** evolutionary history, comparative genomics, wine fermentation, domestication, horizontal transfer, hybrids

## Abstract

*Saccharomyces cerevisiae* and related species, the main workhorses of wine fermentation, have been exposed to stressful conditions for millennia, potentially resulting in adaptive differentiation. As a result, wine yeasts have recently attracted considerable interest for studying the evolutionary effects of domestication. The widespread use of whole-genome sequencing during the last decade has provided new insights into the biodiversity, population structure, phylogeography and evolutionary history of wine yeasts. Comparisons between *S. cerevisiae* isolates from various origins have indicated that a variety of mechanisms, including heterozygosity, nucleotide and structural variations, introgressions, horizontal gene transfer and hybridization, contribute to the genetic and phenotypic diversity of *S. cerevisiae*. This review will summarize the current knowledge on the diversity and evolutionary history of wine yeasts, focusing on the domestication fingerprints identified in these strains.

## Abstract (precis)

This review summarizes current knowledge and recent advances on the diversity and evolutionary history of *Saccharomyces cerevisiae* wine yeasts, focusing on the domestication fingerprints identified in these strains.

## Graphical abstract

**Graphical Abstract Figure.**
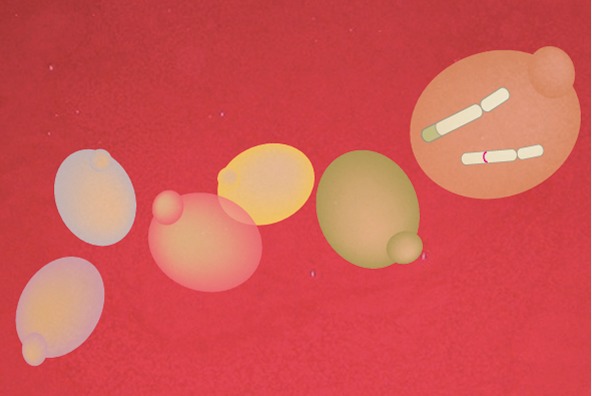
This review summarizes current knowledge and recent advances on the diversity and evolutionary history of *Saccharomyces cerevisiae* wine yeasts, focusing on the domestication fingerprints identified in these strains.

## INTRODUCTION

Although *Saccharomyces cerevisiae* is a well-studied model that has aided our understanding of eukaryotic cellular processes and was the first eukaryotic genome to be completely sequenced (Goffeau *et al.* 1996), this yeast has only recently been established as a model for studies in ecological and evolutionary genetics (Landry *et al.* 2006). Some authors have defended that its closest relative *S. paradoxus* is a better model for ecology and evolutionary biology because it is not associated with humans (Replansky *et al.* 2008). However, *S. cerevisiae* is not domesticated as a whole and population genetics analysis of both domesticated and a growing number of wild isolates is continuously offering new insights into the ecological distribution, population structure and biogeography of this species. In this context, *S. cerevisiae* wine yeasts have attracted considerable interest in recent years.

Wine has a long and rich history, dating back thousands of years, closely associated with the history of agriculture. The earliest archaeological evidence for the production of a fermented beverage made of a mixture of rice, honey and fruit is in China dated to 7000 BC (McGovern *et al.* 2004). The first chemical evidence of the presence of wine is dated to 5400–5000 BC, from the Neolithic period site called Hajji Firuz in Iran, where the presence of combined calcium salt from tartaric acid and terebinth resin was identified in a pottery jar (McGovern, Donald and Glusker 1996). Evidence that *S. cerevisiae* was probably responsible for wine fermentation in Egypt by at least 3150 BC was provided (Cavalieri *et al.* 2003). Wine fermentation technologies expanded from Mesopotamia towards Europe and subsequently spread to the New World (McGovern 2003). Alcoholic fermentation is not only an efficient method for the preservation of the quality and safety of beverages and foods, but wine is also a widespread drug and medicine of antiquity, reflecting the analgesic, disinfectant and preservative properties of this alcoholic beverage. Over time, wine has influenced geography, economics, archeology, history, mythologies and religions, arts and traditions, law and medicine. Today, this beverage has a unique place in most societies, with tremendous economic and cultural value.

It was not until 1860 that Louis Pasteur discovered the origin of fermentation and the implication of yeast (Pasteur 1860). In the early 1880s, Emile Christian Hansen, from the Carlsberg laboratory in Denmark, developed the first pure yeast culture and wort inoculation was performed some years later. In 1890, Müller-Thurgau performed the first inoculation of a grape must with a pure yeast culture. Surprisingly, this practice was effectively used in oenology only in the 1970s, almost one century later. After the 1970s, these practices were generalized and currently most wine fermentations worldwide use selected starter yeasts primarily belonging to *S. cerevisiae*. These practices have improved the control and reliability of the fermentation process, limiting microbiological alterations and have largely contributed to increased wine quality in recent decades. Consistently, most pioneering research in the genetics, genomics, physiology and evolutionary biology of wine yeasts has been developed in *S. cerevisiae* and to a lesser extent on other *Saccharomyces* species and hybrids prominent in the wine industry. This review will focus on the most recent advances on the history, diversity and evolution of *Saccharomyces* wine yeasts.

## *SACCHAROMYCES* AND THE MAKE-ACCUMULATE-CONSUME STRATEGY

The fermentation of grape musts can spontaneously occur through the activity of various microorganisms naturally present on grape berries. More than 40 yeast species have been identified from grape must (reviewed in Jolly, Varela and Pretorius 2014), with the most frequent yeast being from the genera *Hanseniaspora (Kloeckera)*, *Candida, Pichia, Rhodotorula, Debaryomyces*, *Metschnikowia, Kluyveromyces, Schizosaccharomyces, Torulaspora, Zygosaccharomyces* and *Dekkera*. A sequential succession of these yeast species is observed during the early phase of spontaneous fermentation, followed by the disappearance of the majority of them, even if certain non-*Saccharomyces* yeasts persist longer (Fleet, Lafon-Lafourcade and Ribereau-Gayon 1984). This phenomenon might reflect several factors, such as their low fermentative capacity, low tolerance to oxygen limitation and high concentrations of SO_2_ and ethanol. Ethanol-tolerant species, such as *Zygosaccharomyces bailii* (Martorell *et al.* 2007), have been identified throughout fermentation. Non-*Saccharomyces* species might contribute positively or negatively to the organoleptic characteristics of wines (Fleet 1993; Jolly, Varela and Pretorius 2014). Nevertheless, even during spontaneous fermentation, *S. cerevisiae* dominate fermentation and is the primary species responsible for the conversion of sugars to ethanol and CO_2_, reflecting the combination of several ‘winning’ traits, including rapid sugar degradation, ethanol production, accumulation and tolerance, and anaerobic propagation.

One of the most remarkable characteristics of *S. cerevisiae* and closely related species is their ability to produce and accumulate ethanol, referred to as the Crabtree effect, even under aerobic conditions. The long-term Crabtree effect has been explained as a limited respiratory capacity reflecting the repression of respiratory genes (Postma *et al.* 1989; Alexander and Jeffries 1990). However, the immediate occurrence of alcoholic fermentation after the addition of sugar to sugar-limited and respiratory cultures, called the short-term Crabtree effect, has been attributed to an overflow in sugar metabolism (Pronk, Steensma and Van Dijken 1996; Vemuri *et al.* 2007). It has recently been suggested that overflow metabolism is the fundamental mechanism behind both long- and short-term Crabtree effect, which originated approximately 125–150 million years ago (Mya) in the *Saccharomyces* lineage (Hagman and Piškur 2015). Overflow metabolism was first acquired, providing a general strategy to increase energy production rates and enabling rapid glucose consumption. The glucose-mediated repression of respiration would have been acquired as a second step to further increase overflow and ethanol production, thereby inhibiting the growth of other microbes. This characteristic is primarily confined among *S. cerevisiae* and closely related species that diverged after whole-genome duplication, less than 100 Mya (Hagman and Piškur 2015).

*Saccharomyces* and *Dekkera* are characterized by a ‘make-accumulate-consume’ strategy, as these yeasts also efficiently catabolize ethanol. This strategy could confer an advantage to these species in nature, as these yeasts rapidly consume a high quantity of sugars, transforming these carbohydrates into ethanol, which inhibits the growth of other species, and subsequently consuming ethanol after establishing competitive dominance within the ecological niche (Thomson *et al.* 2005; Piškur *et al.* 2006; Rozpędowska *et al.* 2011; Dashko *et al.* 2014).

The make-accumulate-consume strategy emerged after the split between the *S. cerevisiae* and *Kluyveromyces lactis* lineages approximately 100 Mya, suggesting that this process reflected the appearance of modern plants with fruits, which occurred >125 Mya, far earlier than the human domestication of yeast (Thomson *et al.* 2005; reviewed in Piškur *et al.* 2006). Comparative genomics approaches revealed that at least two mechanisms might be involved in the acquisition of this capacity. Thomson *et al.* (2005) reconstructed an ancestral *Saccharomyces* alcohol dehydrogenase gene (*ADH*) and showed that the pre-duplication enzyme was optimized to produce (not consume) ethanol. The make-accumulate-consume strategy emerged with the duplication of *ADH1* and *ADH2*, which occurred after whole-genome duplication approximately 100 Mya (Wolfe and Shields 1997; Kellis, Birren and Lander 2004). The duplication of other genes controlling the flux from hexose to ethanol might have also contributed to the emergence of this strategy (Thomson *et al.* 2005; Conant and Wolfe 2007). Another mechanism to achieve ethanol accumulation involves the global rewiring of the transcriptional network after whole-genome duplication in the *S. cerevisiae* lineage, resulting in the massive loss of regulatory elements from genes involved in respiration (Ihmels *et al.* 2005). Interestingly, the *Dekkera bruxellensis* lineage also lost these specific elements, contributing to the observed Crabtree effect (Rozpędowska *et al.* 2011).

## WINE FERMENTATION: A CHALLENGING ENVIRONMENT

**Figure 1.**
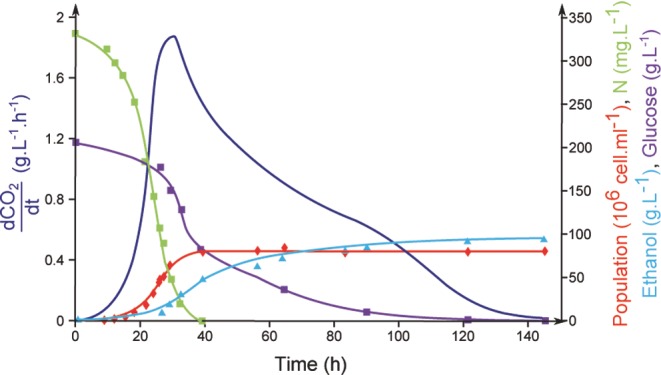
Main phases of wine fermentation. Evolution of the main fermentation parameters during wine fermentation on a synthetic medium containing 200 g L^−1^ glucose/fructose and 330 mg L^−1^ assimilable nitrogen, with the commercial wine strain EC1118 at 24°C. Dark blue: fermentation rate; light blue: ethanol; red: cell number; green: nitrogen; and purple: sugars.

Wine fermentation is a fluctuating environment that exposes yeast to a variety of stresses, including high osmolarity, reflecting increased sugar concentrations, high sulfite levels, anaerobiosis, acid stress, nutrient (nitrogen, lipids and vitamins) depletion and ethanol toxicity. A typical wine fermentation (Fig. 1) comprises a lag phase, which lasts for several hours, a short growth phase of 24–36 h, followed by a stationary phase, during which most of the sugar (between 50 and 80%) is fermented. During this phase, yeast activity continually decreases, although the viability levels remain high, generally over 90%, until the sugar is exhausted. The most desirable traits of wine yeasts include the rapid and complete degradation of sugars into ethanol and CO_2_ to provide metabolites and aroma compounds that positively impact the sensory balance of wine, without producing undesirable compounds (Pretorius 2000; Dequin 2001). Numerous fermentative by-products (glycerol, carboxylic acids, aldehydes, higher alcohols, esters, carbonyl compounds, sulfur compounds, etc.) are derived from the degradation of sugars, amino acids and fatty acids, and yeasts can also transform grape precursors to release varietal aromas (monoterpenes and thiols) (Swiegers *et al.* 2005).

The outcome of fermentation depends on many factors, particularly the amount and quality of nutrients available in grape musts. The primary constituents of grapes must include sugars (glucose and fructose in equimolar amounts, present in high concentrations, 180–300 g L^−1^), organic acids (tartaric and malic), mineral cations (especially potassium), nitrogen compounds and lipids (phytosterols). As yeasts preferably metabolize glucose rather than fructose, fructose is the major sugar present during the late stages of wine fermentation. Wine yeasts must ferment this non-preferred sugar after long periods of starvation and in the presence of large amounts of ethanol. These stressful conditions might alter yeast activity, frequently resulting in sluggish or stuck fermentations (Bisson 1999; Bauer and Pretorius 2000). The ability of wine yeasts to ferment fructose is therefore critically important for the maintenance of a high fermentation rate at the end of alcoholic fermentation.

Nitrogen is an important nutrient present in limited amounts in grape musts, and the availability of this nutrient is directly associated with biomass production, which governs the fermentation rate and production of volatile compounds. Consequently, nitrogen deficiency is the most prevalent cause of stuck and sluggish fermentations (Bisson 1999; Bell and Henschke 2005; Sablayrolles 2008). Yeast assimilable nitrogen (YAN) primarily comprises ammonium ions and amino acids (Henschke and Jiranek 1993). However, other nitrogen sources, e.g. oligopeptides, polypeptides, proteins, amides, biogenic amines and nucleic acids, might constitute substantial nitrogen resources (Ough, Huang and Stevens 1991; Henschke and Jiranek 1993). It is generally considered that 140 mg L^−1^ of YAN is necessary to complete the fermentation of 200 g L^−1^ sugar, whereas approximately 300 mg L^−1^ of YAN is required to optimize fermentation kinetics (Sablayrolles 2008).

Lipids are also key nutrients in alcoholic fermentation. The synthesis of sterols and unsaturated fatty acids requires oxygen. Limited amounts of unsaturated fatty acids or sterols negatively affect viability at the end of fermentation (Alexandre, Rousseaux and Charpentier 1994; Luparia *et al.* 2004). The strong clarification of grape musts leads to lipid limitation typically associated with a loss of yeast cell viability. Recent studies have shown that the nitrogen status of grape musts is a strong determinant of the outcome of alcoholic fermentations under conditions of lipid limitation (Tesnière *et al.* 2013). Other important nutrients are vitamins such as thiamin, which is rapidly consumed by yeasts. Thus, indigenous yeasts might cause thiamin depletion in grape musts, with negative consequences for the inoculation efficiency and fermentation kinetics (Bataillon *et al.* 1996). In addition, various inhibitors, including mid-length chain fatty acids, killer toxins or SO_2_ which is frequently used in grape juice as antimicrobial or antioxidant, might affect yeasts during wine fermentation. However, ethanol is widely recognized as one of the causes of stuck or sluggish alcoholic fermentation (Bisson 1999). Ethanol exerts a biological effect on growth and fermentation efficiency, reflecting an increase in membrane permeability and changes in membrane fluidity (Alexandre, Rousseaux, Charpentier 1994). Wine yeasts are also exposed to a wide range of temperatures, as red wine fermentation is generally conducted at high temperatures (l8–25°C) and white wine fermentation typically occurs at cooler temperatures (l0–15°C).

Thus, for millennia, winemaking conditions have exposed yeasts to a combination of stresses (osmotic, acidic, nutrient starvation, ethanol toxicity) acting individually or synergistically, potentially leading to adaptive differentiation.

## DIVERSITY AND EVOLUTIONARY HISTORY OF *S. CEREVISIAE* WINE STRAINS

### Genetics and life style

*Saccharomyces cerevisiae* has a haplo-diploid life cycle and a life history predominantly involving clonal reproduction. The frequency of outcrossing remains a matter of debate. The outcrossing rate was initially considered a rare event, occurring once every 50 000 divisions (Ruderfer *et al.* 2006). However, a recent study, based on different methods, estimated a considerably higher rate of approximately 1 per 100 mitotic divisions (Kelly *et al.* 2012). Outcross matings have been estimated at 2% and 36–52% for oaks and domesticated isolates (Magwene *et al.* 2011), and 8–20% for vines and wines strains (Gayevskiy and Goddard 2012). Wine yeasts also have a low level of linkage disequilibrium (falls to one half of is maximal value in approximately 2.5 kb) compared with other isolates, likely reflecting a higher frequency of outcrossing events (Schacherer *et al.* 2009). These data suggest that human-associated environments might create greater opportunities to bring diverse strains into proximity (Goddard *et al.* 2010) or for spore dispersal through insect vectors (Reuter, Bell and Greig 2007). Indeed, Reuter, Bell and Greig (2007) suggested that outcrossing would be more effective after the partial digestion of asci by *Drosophila*.

Most *S. cerev*isiae strains isolated from the environment, including vineyards, are diploid cells (Cubillos *et al.* 2009). Wine isolates are primarily homothallic (Mortimer 2000), producing haploid spores that switch mating types and undergo self-diploidization. The pioneering studies of Robert Mortimer showed that homothallic isolates are generally heterozygous at one or more loci, and the frequency of heterozygosity was negatively correlated with the viability of the spores produced from the vineyard isolates. This heterozygosity in homothallic isolates has been attributed to both mutations that occur during the mitotic growth of homozygous diploid isolates (Mortimer *et al.* 1994; Johnston, Baccari and Mortimer 2000) and the outcrossing of homothallic isolates (McCusker 2006). This finding led Mortimer to propose the ‘Genome Renewal Hypothesis’ (Mortimer *et al.* 1994; Mortimer 2000), suggesting that recessive, deleterious heterozygous mutations accumulate during mitotic growth and are subsequently eliminated through rare sexual cycles involving meiosis, followed by mating-type switching and autodiploidization. Thus, deleterious alleles would be lost, and beneficial alleles would be fixed, thereby facilitating adaptation in yeast. Recent data obtained through the whole-genome sequencing of diploid isolates revealed a more extensive level of heterozygosity than initially considered, particularly in domesticated isolates (Magwene *et al.* 2011). The number of heterozygous single nucleotide polymorphisms observed in the genome of commercial wine yeasts ranged from 1000 to more than 18 000 (Novo *et al.* 2009; Borneman *et al.* 2011). The high levels of heterozygosity reflect population admixture due to human domestication, coupled with selfing during rare sexual cycles, and these effects may facilitate rapid adaptation to novel environments by increasing the genetic and phenotypic diversity in the population deriving from a single isolate (Magwene 2014). Thus, sexual reproduction and outcrossing are rare but important features in the *Saccharomyces* life cycle.

### Wine yeast strains have a unique origin

In the last decade, numerous studies, based on multigene sequencing (Fay and Benavides 2005; Stefanini *et al.* 2012; Wang *et al.* 2012), microsatellite analysis (Legras *et al.* 2007), tiling array hybridization (Schacherer *et al.* 2009), low coverage whole-genome sequencing (Liti *et al.* 2009) or restriction-site-associated sequencing (Rad-seq) (Cromie *et al.* 2013) have provided deep insight into the population structure and evolutionary history of *S. cerevisiae*. Domesticated strains of *S. cerevisiae*, particularly those used for the production of sake and wine, were derived from the natural population through independent domestication events (Fay and Benavides 2005; Legras *et al.* 2007). Legras *et al.* (2007) showed that 95% of strains associated with wine belong to the same cluster, suggesting a unique origin of wine yeasts, followed by the expansion of populations through human activities.

From a set of *S. cerevisiae* isolates with worldwide origin, five distinct lineages were revealed based on their technological and geographic origin (West African, Malaysian, North American, Sake and European/wine), and many strains with mosaic genomes resulting from crosses between these well-defined lineages were identified (Liti *et al.* 2009). Using another set of strains, Schacherer *et al.* 2009 identified three distinct lineages (wine, sake and laboratory), which reflect different ecological origins. A recent genomic survey of a higher number of strains suggested a model of geographic differentiation, followed by human-associated admixture, primarily occurring between European and Asian populations and more recently between European and North American populations (Cromie *et al.* 2013). In addition to global-scale pictures, several studies have investigated the structure and gene flow at ecological levels. Analyses of vineyard isolates have provided evidence for region-specific subpopulations (Gayevskiy and Goddard 2012; Schuller *et al.* 2012), consistent with previous observations (Legras *et al.* 2007). Evidence of gene flow across small distances between populations inhabiting vineyards and distinct oak tree populations has also been reported, suggesting some degree of connectivity between populations (Hyma and Fay 2013; Knight and Goddard 2015).

These studies showed that *S. cerevisiae* as a whole is not domesticated and that the population structure of this species, at least partially, reflects different ecological niches. Specifically, wine strains form a distinct phylogenetic group, with low diversity (Fay and Benavides 2005; Legras *et al.* 2007; Liti *et al.* 2009; Schacherer *et al.* 2009; Cromie *et al.* 2013). The diversity between wine strains has been estimated as 1 to 1.4 substitutions per kb and 5 to 6 substitutions per kb between wine and other *S. cerevisiae* strains from other origins (Fay and Benavides 2005; Liti *et al.* 2009; Schacherer *et al.* 2009).

A microsatellite-based study suggested that wine yeast strains could originate from Mesopotamia (Legras *et al.* 2007; Sicard and Legras 2011). Two migration routes could have led the yeasts into Europe: the first route through the Mediterranean Sea to Italy, France and Spain, and in France from the Mediterranean coast to Burgundy through the Rhone valley, and the second route through the Danube valley. Yeast strains could have also been transferred via comigration with grape varieties (Sicard and Legras 2011). The identification of three Chinese wild isolates belonging to the Wine/European lineage by Wang *et al.* (2012) led these authors to raise the possibility that Wine/European strains have an Asian origin, in line with previous archaeological evidence for fermented beverage in China dated to 9000 years ago (McGovern *et al.* 2004). However, these isolates were sampled from orchard soil and grape and might not be truly natural. The opposite hypothesis stipulating that the wine yeast could have migrated to Asia is also plausible.

### Phenotypic divergence between wine and non-wine strains

There is strong evidence of marked phenotypic divergence between wine and non-wine strains. Wine yeasts strains demonstrate better resistance to copper (Fay *et al.* 2004; Warringer *et al.* 2011) and sulfites (Park and Bakalinsky 2000; Pérez-Ortín *et al.* 2002), two chemical compounds used in vineyards and during winemaking. Several wine yeasts strains have also demonstrated an ability to utilize xylose as a carbon source (Wenger, Schwartz and Sherlock 2010) or different types of ditripeptides as nitrogen sources (Homann *et al.* 2005). Liti *et al.* (2009) reported a correlation between strain genetic diversity and growth in different environments or in the presence of drugs. The Wine/European lineages and mosaics showed rapid growth compared with other lineages, and this feature could be advantageous for fermentation processes in which many of these strains are used. There are few large-scale studies comparing the performance and metabolite production of strains under relevant conditions for fermentation. Divergence in life-trait strategies has been reported between industrial strains, referred to as extreme ‘grasshoppers’, which reproduce slowly, display a small carrying capacity and have a large cell size compared with natural and laboratory isolates, referred to as ‘ants’, which reproduce rapidly, display a large carrying capacity and have a small cell size (Spor *et al.* 2009).

**Figure 2.**
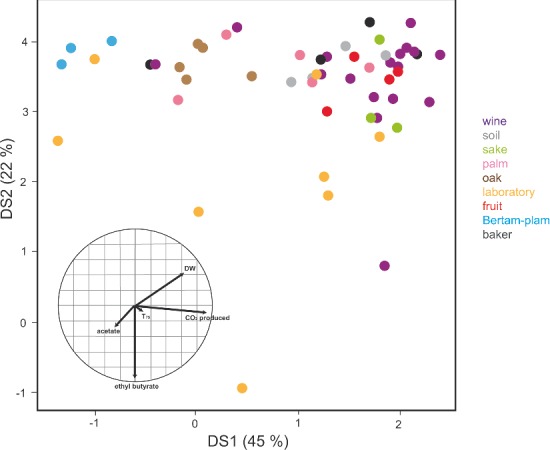
Ability of *S. cerevisiae* from various origins to ferment large amounts of sugar under winemaking conditions. Linear discriminant analysis (LDA) of the population based on dry weight, 75% sugar fermentation, CO_2_ production, acetate and ethyl butyrate production of 53 strains during fermentation in a synthetic must (adapted from Camarasa *et al.* 2011).

The comparison of 72 strains of various origins in wine fermentation conditions revealed substantial variations in fermentative properties and growth and metabolite traits. Wine strains and other strains isolated from sugar-rich environments (fruits) had a better fermentative capacity under oenological conditions than natural strains isolated from sugar-poor environments (Camarasa *et al.* 2011) (Fig. 2). Another study, based on a restricted number of strains and on sensorial analysis, suggested that wine yeasts produce higher fruity aromas in wine compared with strains from other origins (Hyma *et al.* 2011). The analysis of a higher number of strains representing each group and under conditions simulating different habitats is needed to obtain a better picture of the adaptation of these strains to specific conditions.

*Saccharomyces cerevisiae* traits might be a consequence of genetic drift rather than selection (Warringer *et al.* 2011). For example, the West-African population is phenotypically unique, with an extreme abundance of low-performance alleles. However, domestication traits likely reflect both genetic drift and human selection of specific traits (Warringer *et al.* 2011). In the following section, we will describe compelling evidence and cases of adaptive evolution in wine yeasts.

## EVIDENCE FOR HUMAN-ENFORCED ADAPTIVE EVOLUTION

Yeasts use several mechanisms to respond to environmental challenges and evolve corresponding adaptive functions. Adaptation can be achieved through small-scale nucleotide changes (base insertions, deletions or substitutions), which alter protein structure, protein interactions or gene expression, large-scale genome rearrangements (chromosome duplications, translocations and aneuploidy), which alter gene expression through the modification of the genomic context, or copy number variations (CNV), which might alter the gene dosage. In addition, introgression and horizontal transfer could exert powerful evolutionary forces by generating novelties that cannot be introduced using other nucleotide changes or structural rearrangements.

These mechanisms have been shown to contribute to the adaptation of wine yeast genomes (Pretorius 2000; Barrio *et al.* 2006; Blondin *et al.* 2009; Dequin and Casaregola 2011).

### Hallmarks of domestication in Flor strains

An interesting case of traits acquired after human domestication has been reported in flor yeast. Flor strains form a biofilm on the surface of wine after fermentation and develop oxidative metabolism in the presence of a high concentration of ethanol and a low level of fermentable sugar, i.e. fructose (Alexandre 2013). Because flor yeasts continuously grow on the surface of wine during the sherry wine making, the life style of these microorganisms is completely different from that of fermentative *S. cerevisiae* wine yeasts, which makes these strains an interesting model to study evolution. The acquisition of two mutations in the promoter and coding regions of the *FLO11* gene encoding a GPI-anchored cell surface glycoprotein (flocculin) required for pseudohyphal formation, invasive growth, flocculation and biofilm formation (Guo *et al.* 2000; Fidalgo *et al.* 2006) results in increased *FLO11* expression and enhanced cell adhesion. A study in a fructophilic wine yeast strain (Guillaume *et al.* 2007) identified a natural allelic variant of *HXT3* encoding a major glucose transporter during wine fermentation, which enhances fructose fermentation. This allele is frequently found in flor strains (Coi A, Dequin S, Legras JL, unpublished data), which might be related to a better capacity of these strains to use fructose. Interestingly, a population study of flor yeasts using microsatellite analyses recently showed that these strains form a unique group, closely related to wine yeasts (Legras, Erny and Charpentier 2014). Comparative genomics between these two close but distinct groups with contrasting life styles offers promising perspectives to identify traces of selection in this group.

### Adaptation of wine yeasts to chemicals used in vineyards

The existence of gross chromosomal rearrangements (GCR), i.e. translocations, deletions and amplifications of chromosomal regions, was proposed in the 1990s based on the high level of chromosome polymorphism observed in wine yeasts (Vezinhet, Blondin and Hallet 1990; Yamamoto *et al.* 1991; Bidenne *et al.* 1992; Codón, Benítez and Korhola 1998). These GCR events are mediated through ectopic recombination between repeated Ty sequences or other repeated sequences (Rachidi, Barre and Blondin 1999). Several chromosomal translocations have been identified in wine yeast genomes, particularly in telomeric regions, consistent with the idea that peripheral regions are highly plastic (Borneman *et al.* 2008, 2011; Novo *et al.* 2009). In most cases, there is no evidence that these rearrangements contribute to yeast fitness.

**Figure 3.**
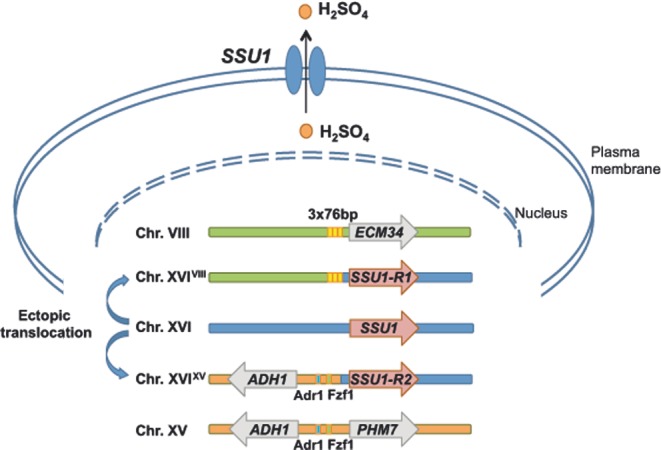
Mechanisms of sulfite resistance through reciprocal translocations. Two ectopic translocations resulting in increased expression of *SSU1*, encoding a plasma membrane sulfite pump enabling yeast cells to resist to sulfite have been described. A first translocation (VIII-t-XVI, the most frequent form) was mediated through crossing-over mediated by microhomology within the promoters of *ECM34* and *SSU1*. Several 76-bp (in yellow) repeats (3–6 tandem repeats) were found in the promoters of non-recombinant ECM34 and recombinant SSU1-R1. A direct relationship between the number of 76-bp repeats and sulfite tolerance has been described (Pérez-Ortín *et al.* 2002). A second reciprocal translocation (XV-t-XVI) involves Adr1 (blue rectangle) and Fzf1 (green rectangle) binding regions of the promoter of *ADH1* and *SSU1*, respectively, resulting in the SSU1-R2 allele having increased expression during the first hours of alcoholic fermentation (Zimmer *et al.* 2014).

A well-documented example of chromosomal rearrangement with an adaptive advantage is the reciprocal translocation between chromosome VIII and XVI, which is widespread among wine yeasts. This translocation generated a dominant allele of the sulfite pump, *SSU1-R1*, which is expressed at much higher levels than *SSU1* (Goto-yamamoto *et al.* 1998) and confers a high level of sulfite resistance (Goto-Yamamoto 1998; Pérez-Ortín *et al.* 2002; Yuasa *et al.* 2004). Recently, another translocation between chromosome XV and XVI was identified in several wine strains through quantitative trait loci (QTL) mapping for lag phase duration in the alcoholic fermentation of grape juice, and this translocation increased the expression of this gene (Zimmer *et al.* 2014). The VIII-t-XVI and XV-t-XVI translocations (Fig. 3) have only been observed in wine yeasts, and 88% of 36 wine strains analyzed possess at least one of these translocations. The VIII-t-XVI translocation is the more frequent and the XV-t-XVI form was only found in commercial starter wine strains, suggesting a recent event. Both translocations conferred a selective advantage by shortening the growth lag phase in medium containing SO_2_. Thus, the wide use of sulfites since the Middles Ages (Pérez-Ortín *et al.* 2002) likely caused an evolutionary bottleneck, favoring convergent evolutionary rearrangements that confer a growth advantage to strains carrying the *SSU1* recombinant forms.

Another potential domestication-related trait is the acquisition of resistance to copper sulfate. Elevated copper tolerance in the European and Sake lineages has been associated with a CNV of *CUP1* encoding the copper-binding metallothionein (Warringer *et al.* 2011). The emergence of the *CUP1* CNV in these lineages, but not in other *S. cerevisiae* populations or in *S. paradoxus*, strongly suggests that the *CUP1* CNV reflects convergent evolution due to human selection for industrial production (Warringer *et al.* 2011). Consistent with a previous study using a restricted number of strains (Fay *et al.* 2004), recent studies based on a higher number of strains revealed increased resistance to CuSO_4_ associated with a higher number of copies of *CUP1* among wine strains compared with oak or other isolates (Almeida *et al.* 2015). These data suggest that the acquisition of this trait could be associated with the use of copper sulfate in vineyards, which has been used as a fungicide against powdery mildew since the 1880s (Fay *et al.* 2004).

Recently, a promoter variant of *CUP1* with increased expression variability was identified in the wine yeast strain EC1118. This promoter provides a benefit under environmental stress conditions, suggesting that modulation of gene expression is another potential adaptation mechanism in yeast (Liu *et al.* 2015).

In addition to these examples, genome-wide studies have provided more complete insights into structural variation, revealing the existence of many CNV in wine yeasts, corresponding to genes encoding transporters or dehydrogenases or genes involved in drug response (Dunn, Levine and Sherlock 2005; Carreto *et al.* 2008; Borneman *et al.* 2011; Warringer *et al.* 2011).

### Adaptive loss of aquaporins in wine yeasts

The water transporters aquaporins *AQY* are critical for surviving freeze–thaw stress. It was suggested that rapid export of water increases freeze–thaw survival by preventing intracellular shearing due to water crystallization (Tanghe *et al.* 2002). On the other hand, Wills *et al.* (2010) showed that loss of *AQY*s function provides a major fitness advantage on high-sugar substrates to overcome the effect of high osmolarity. Laboratory and industrial strains as well as several vineyard isolates harbor non-functional alleles of *AQY2* while several strains also harbor a non-functional version of *AQY1* (Bonhivers *et al.* 1998; Laizé *et al.* 2000). These paralogs have been lost at least six independent times through missense and frame-shift mutations (Will *et al.* 2010). However, Malysian strains which are not associated with domestication events show unique non-functional *AQY* alleles, indicating that loss of aquaporins is not strictly driven by domestication. The antagonistic effect of *AQY*s contributes to the maintenance of both functional and nonfunctional alleles in *S. cerevisiae*.

### Introgressions from *Saccharomyces* sp. in wine yeasts

Several *S. paradoxus* and *S. mikatae* introgressions were identified in *S. cerevisiae* wine strains (Dunn *et al.* 2012). A large *S. paradoxus* introgressed region, identified in commercial wine yeast strains, spans a region corresponding to the *SUC2* region of the *S. cerevisiae* genome and includes not only the *S. paradoxus SUC2* gene, which encodes sucrose-hydrolyzing invertase, but also a gene similar to *S. cerevisiae HPF1*, encoding a glucan alpha-1,4-glucosidase that, when overexpressed, reduces protein haze formation in white wines (Brown *et al.* 2007). Furthermore, this introgressed region also contains *AWA1*, a gene present in *S. cerevisiae* sake strains but absent from S288C, encoding a putative GPI-anchored protein localized to the cell wall and conferring hydrophobicity to the cell surface for foam formation in sake mash (Miyashita *et al.* 2004). This evidence suggests that some adaptive or industrially desirable qualities might be conferred by *S. paradoxus* genes to these wine strains (Dunn *et al.* 2012).

In addition to *S. cerevisiae*, the cryotolerant species, *S. uvarum*, is also used for wine and cider fermentation. A recent study of the population structure and diversity of this species revealed multiple introgressions from other *Saccharomyces* species, and those from *S. eubayanus* were prevalent in European strains associated with human-driven fermentations (Almeida *et al.* 2014). This study suggests that the anthropic habitats colonized by *S. uvarum* in Europe might have favored the hybridization of *S. uvarum* with *S. eubayanus*, followed by subsequent introgression through backcrossing to *S. uvarum*. These introgressed regions are enriched in functions involving nitrogen metabolism, suggesting that these regions might confer an advantage under nitrogen-limiting wine fermentation conditions.

### Horizontal transfer and evolutionary advantage of *FOT* genes

In the last decade, comparative genomics revealed the previously unsuspected contribution of horizontal gene transfer (HGT) to the adaptation of wine yeasts. The genome of the commercial *S. cerevisiae* wine yeast EC1118 unexpectedly contained three large chromosomal segments, A, B and C (120 kb in total), acquired through independent HGT events from distant yeast species (Novo *et al.* 2009). These regions have primarily been identified in wine yeasts and mosaic genomes (Borneman *et al.* 2008, 2011; Novo *et al.* 2009). *Zygosaccharomyces bailii*, a major contaminant of wine fermentations, was identified as the donor of region B (Novo *et al.* 2009; Galeote *et al.* 2011). Multiple copy insertions and different arrangements of region B have been identified in various wine strains, suggesting that a circular intermediate is involved in the amplification and expansion of this region (Borneman *et al.* 2011; Galeote *et al.* 2011). Recently, Marsit *et al.* (2015) showed that region C also results from a recent transfer, dated approximately 2000 years ago, from *Torulaspora microellipsoides*, a distant yeast species identified in the wine environment. Thus, recurring transfer from distant yeasts have shaped the genome of wine yeasts, indicating the evolutionary advantage and biological relevance of HGT genes.

The three initially identified large genomic regions comprise 39 genes (including 5 pseudogenes) encoding functions potentially important for winemaking, such as sugar and nitrogen metabolism (Novo *et al.* 2009). The functions of several genes of the *T. microellipsoides* region were characterized in detail. For example, *FSY1* encodes a high-affinity fructose/H^+^ symporter that might be advantageous at the end of wine fermentation, when fructose is the most abundant sugar (Galeote *et al.* 2010). Another gene, *XDH1*, encodes a putative xylitol dehydrogenase involved in xylose metabolism (Wenger, Schwartz and Sherlock 2010). Two other tandem duplicated genes *FOT1–2* encode oligopeptide transporters, which considerably increase the range of oligopeptides typically transported by the carrier proteins Ptr2p and Dal5p in *S.* *cerevisiae* (Damon *et al.* 2011).

**Figure 4.**
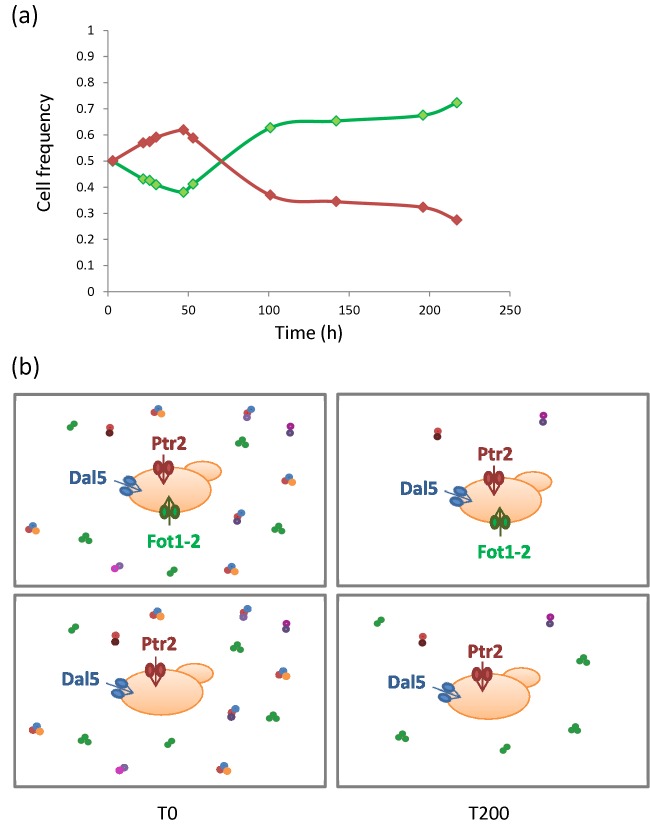
Competitive advantage of *FOT* genes acquired through HGT in wine yeasts during grape must fermentation. (**a**) Frequency of cocultured wine strains with (green) or without (red) *FOT* genes labeled with different fluorochromes and monitored through flow cytometry during fermentation. (**b**) Wine strains with *FOT* genes use a broader range of the oligopeptides present in grape juice, particularly those rich in glutamate (colored in green), compared with strains without *FOT* genes (Marsit *et al.* 2015).

Comparative genomics has provided new insights into the evolutionary history of region C. This region is widespread among wine strains and underwent several rearrangements, including gene losses and gene conversion through *FOT* genes, resulting in a patchy distribution among various strains (Marsit *et al.* 2015). *FOT1–2* genes are strongly conserved among region C genes, which suggest that they might have an evolutionary advantage. Using competition experiments, Marsit *et al.* (2015) demonstrated that the presence of *FOT* genes provides a strong competitive advantage on a natural grape must (Fig. 4). These genes facilitate the transport of a broader range of oligopeptides present in grape juice, particularly those rich in glutamate, which are the most abundant, resulting in improved biomass formation, fermentation efficiency and cell viability during winemaking (Marsit *et al.* 2015). Furthermore, Fot-mediated peptide uptake substantially affects the central pathways of carbon and nitrogen metabolism, amino acid and protein biosynthesis and the oxidative stress response. In particular, the glutamate node and the NADPH/NADP^+^ balance are markedly modified, resulting in decreased acetic acid production and increased ester formation, which might improve the organoleptic balance of wines (Marsit S, Galeote V and Dequin S, unpublished data). In addition, several *FOT* alleles, generated through gene conversion from the *FOT* genes of *T. microellipsoides*, were identified in wine yeasts. These variants might have acquired potentially specialized functions, which remain uncharacterized.

Interestingly, the ability to use different types of ditripeptides as nitrogen sources considerably varied from one strain to another (Homann *et al.* 2005). The presence of *FOT* genes likely contributes to this phenotypic variation in *S. cerevisiae*.

In addition to *FOT* genes, several genes present in new regions acquired by wine yeasts have putative functions associated with nitrogen metabolism, including asparaginase, oxoprolinase, an ammonium transporter, an allantoate transporter and two transcription factors associated with the biosynthesis enzymes involved in lysine and proline utilization (Novo *et al.* 2009). Several introgressions from *S. eubayanus* in *S. uvarum* wine strains also contain genes involved in nitrogen metabolism (exopeptidase, L-asparaginase) (Almeida *et al.* 2014). These genes might facilitate the utilization of nitrogen resources, which is limiting in grape must, providing a competitive advantage to wine yeasts for nutrients during winemaking. These data suggest the concerted evolution of the genome of wine yeasts associated with nitrogen metabolism. Consistently, it was recently suggested that life stage performances have evolved in concert with nitrogen use (Ibstedt *et al.* 2015).

Little is known about the mechanisms at the origin of an introgression between two different yeast species. In plants, introgressions frequently result from hybridization, followed by successive backcrossing. In nature, the succession of backcrosses seems unlikely, considering the limited frequency of the yeast sexual cycle compared with clonal division, according to Ruderfer *et al.* (2006). In addition, the sequence divergence between the different species increases the number of necessary backcrosses because this phenomenon reduces the frequency of meiotic recombination. Other mechanisms have been suggested, such as the unidirectional transfer of a single chromosome, chromosome fragment or an episome from one nucleus to another in a newly formed hybrid prior to karyogamy (Morales and Dujon 2012). An alternative explanation is hybridization followed by the loss of most chromosomal material of one of the parents. Many artificial hybrids have been constructed so far, including from distantly related yeasts (Morales and Dujon 2012). These studies showed that hybrid lines generally undergo progressive genome stabilization, during which large genomic rearrangements occur, including aneuploidization, chromosomal translocation and partial or total chromosome loss. Marinoni *et al.* (1999) tried to obtained interspecific hybrids by crossing yeasts belonging to the genus *Saccharomyces*, including species of the former sensu stricto and sensu lato groups. They observed that in the case of more distantly related parents, the frequency of interspecific zygotes was lower and that only one parental set, and perhaps some fragments of the other one, could be found in genetically stabilized hybrid lines. Anticipating later findings, they concluded that if *Saccharomyces* isolates could mate freely in nature, horizontal transfer of genetic material could have occurred during the evolution of modern yeast species.

### Wine yeast hybrids

Interspecific hybridization provides new combinations of genes and might confer selective advantages over the parental species (Masneuf *et al.* 1998; Dujon 2010; Morales and Dujon 2012). In recent decades, a growing number of natural hybrids between two or more *Saccharomyces* species have been identified in yeast. The best known example is the brewing yeast *S. pastorianus*, a hybrid between *S. cerevisiae* and *S. eubayanus* (Libkind *et al.* 2011). The molecular characterization of wine and cider yeasts also revealed many hybrids formed independently between *S. cerevisiae/S. kudriawzevii* (Bradbury *et al.* 2006; González *et al.* 2006; Lopandic *et al.* 2007; Sipiczki 2008; Arroyo-López *et al.* 2009; Gangl *et al.* 2009; Borneman *et al.* 2012; Erny *et al.* 2012), *S. cerevisiae/S. uvarum* (Masneuf *et al.* 1998; Masneuf *et al.* 2002; Le Jeune *et al.* 2007; Sipiczki 2008) or between *S. cerevisiae/S. kudriawzevii/S. uvarum* (Naumova *et al.* 2005; González *et al.* 2006).

Hybrids might present several advantages over non-hybrids in wine fermentation (González *et al.* 2007; Arroyo-López *et al.* 2009; Gangl *et al.* 2009; Tronchoni *et al.* 2009). These hybrids often show more robust features than the parents, such as tolerance to various stresses during fermentation (Belloch *et al.* 2008; Morales and Dujon 2012). For example, *S. kudriavzevii* and *S. bayanus* are better adapted to growth at low temperatures compared with *S. cerevisiae* wine strains, whereas *S. cerevisiae* is more alcohol tolerant. The natural hybrids between these species have adapted to growth under ethanol and temperature stress through the inheritance of competitive traits from one or another parental species (Belloch *et al.* 2009). In recent years, winemakers have preferred the fermentation of white wines at low temperatures, ranging from 10 to 15°C, to minimize the loss of aromatic volatile compounds. Therefore, these hybrids have potential value under these conditions. These inherited traits might also influence the aromatic complexity of wine. *Saccharomyces cerevisiae × Saccharomyces kudriavzevii* hybrids have been described as greater producers of esters and higher alcohols depending on grape variety (González *et al.* 2007; Lopandic *et al.* 2007; Gangl *et al.* 2009). These hybrids also release much higher amounts of the fruity thiol 4-mercapto-4-methylpentan-2-one from grape-derived non-aromatic precursors than other commercial wine yeast strains (Dubourdieu *et al.* 2006; Swiegers *et al.* 2009). The abundance of these hybrids could reflect an adaptive advantage, but it is also possible that stressful conditions trigger hybridization events (Replansky *et al.* 2008). Hybrid lines generally undergo progressive genome stabilization, during which chromosomal rearrangements and modifications of the genetic contribution of relative parents, aneuploidy or partial chromosome losses were observed (Antunovics *et al.* 2005; Querol and Bond 2009; Kunicka-Styczyńska and Rajkowska 2011; Borneman *et al.* 2012; Morales and Dujon 2012).

The molecular characterization of 24 *S. cerevisiae–S. kudriavzevii* hybrids from Northern European winemaking environments (including commercial strains) revealed multiple ploidy levels (from 2n to 4n) and various amounts of *S. kudriavzevii* genetic content (Erny *et al.* 2012). These strains result from multiple hybridization events between several *S. cerevisiae* wine yeast isolates and various *S. kudriavzevii* strains (Erny *et al.* 2012). Another commercial wine strain, Vin7, is an almost complete allotriploid interspecific hybrid containing a heterozygous diploid *S. cerevisiae* genome and a haploid *S. kudriavzevii* genome with several homologous recombination and genomic substitution between the two genomes (Borneman *et al.* 2012). Both parental strains were of European origin, and the *S. cerevisiae* parent was closely related to, but distinct from, the commercial wine yeasts QA23 and EC1118 (Borneman *et al.* 2012). Strikingly, *S. kudriavzevii* yeast strains have never been isolated from wine fermentation, but have been initially isolated from decaying leaves in Japan. Thus, it is unclear how this species formed the hybrids identified in Europe (Naumov *et al.* 2000). However, recent environmental sampling identified *S. kudriavzevii* in Portugal and France (Ardèche), but associated with oak bark (Sampaio and Gonçalves 2008; Erny *et al.* 2012). Despite a common European origin, it remains unknown where and when *S. cerevisiae* and *S. kudriavzevii* hybridization occurs.

A recent study provided experimental evidence of evolutionary innovations resulting from hybrid formation. Interspecific hybrids between *S. cerevisiae* and *S. uvarum* were *de novo* generated and subjected to experimental evolution under ammonium limitation conditions. A rearranged interspecific fusion of *MEP2*, encoding a high-affinity ammonium permease, was shown to confer enhanced fitness under these conditions (Dunn *et al.* 2013). This rearrangement resulted from the introgression of several bases from *S. uvarum* in chromosome XIV of *S. cerevisiae*. The architecture of *MEP2* rearrangements suggests a rapid introgression model, which does not require repeated backcrossing with the parental species (Dunn *et al.* 2013).

## PERSPECTIVES

Recent advances in genome-wide analyses and next-generation sequencing have provided unprecedented insights into the population structure and evolutionary history of *Saccharomyces*, revealing the impact of yeast domestication. Compelling evidence of adaptation in wine yeast strains has been provided, showing that wine yeasts use a variety of mechanisms, including nucleotide and structural variations, introgressions and HGT, to adapt to the winemaking environment. Expanding the whole-genome sequence dataset of strains from the wine environment and other anthropic niches will provide a better understanding of the evolutionary history of domesticated strains and the frequency of these mechanisms, particularly HGT. In addition, the availability of a higher number of genome sequences might facilitate the identification of allelic variants and other divergent regions involved in the adaptation to wine making, which first genomic population approaches have not been able to detect. For example, flor and wine strains belonging to closely related groups with contrasting lifestyles, such as aerobic respiration versus sugar fermentation, might constitute a relevant model to identify divergent regions that might explain the adaptation to these niches. Although QTL mapping strategies have been successfully used in recent decades to decipher the genotype–phenotype associations among well-defined sets of parental strains, sequence information will also increase the number of variants, enabling genome-wide association strategies.

Despite the clear evidence that wine yeast strains have been selected and domesticated from wild strains and subsequently dispersed, little is known about the ecological life cycle and natural history of *S. cerevisiae*. Indeed, how yeast cells survive in the absence of rich sugar sources in natural environments, particularly during the winter, remains puzzling. A role for birds and insects (Drosophila, bees) as vectors for *S. cerevisiae* has been suggested (Goddard *et al.* 2010; Francesca *et al.* 2012; Buser *et al.* 2014). A recent study demonstrated a role for social wasps as vectors and natural reservoirs for *S. cerevisiae* during all seasons, and these authors suggested a multidirectional flow of *S. cerevisiae* between wineries and vineyards (Stefanini *et al.* 2012). A major challenge in the future will be to understand how these processes influence the observed population patterns. Metapopulation genomics studies quantifying ecological-scale population processes might provide information to increase the current understanding of gene flow between populations (Knight and Goddard 2015).
